# Supplementary material for: Human whole genome sequencing in South Africa
Source: Sci Rep. 2021 Jan 12;11:606. doi: 10.1038/s41598-020-79794-x (PMC7803990; doi:10.1038/s41598-020-79794-x)
Supplement: Supplementary file 1 — Supplementary Information 1. [file 41598_2020_79794_MOESM1_ESM.docx]

**Human Whole Genome Sequencing in South Africa**

Brigitte Glanzmann^1,15#^, Tracey Jooste^2,3,15#^, Samira Ghoor^2,15#^, Richard Gordon^4^, Rizwana Mia^4,15^, Jun Mao^5,15^, Hao Li^5^, Patrick Charls^6^, Craig Douman^6^, Maritha J. Kotze^7,8^, Armand V. Peeters^7^, Glaudina Loots^9^, Monika Esser^10^, Caroline T. Tiemessen^11^, Robert J Wilkinson^12,13,14^, Johan Louw^2^, Glenda Gray^16,17^, Robin M. Warren^1^, Marlo Möller^1,15,#^, Craig Kinnear^1,15, #*^

^1^DSI-NRF Centre of Excellence for Biomedical Tuberculosis Research, SAMRC Centre for Tuberculosis Research, Division of Molecular Biology and Human Genetics, Faculty of Medicine and Health Sciences, Stellenbosch University, Cape Town, South Africa.

^2^Biomedical Research and Innovation Platform, South African Medical Research Council, Tygerberg, Cape Town, South Africa.

^3^Division of Medical Physiology Faculty of Medicine and Health Sciences, Stellenbosch University, Tygerberg Hospital, Cape Town, South Africa.

^4^Grants, Innovation and Product Development, South African Medical Research Council, Tygerberg, Cape Town.

^5^ BGI-Shenzhen- Building 11, Beishan Industrial Zone, Yantian District, Shenzhen (518083).

^6^Information Technology Services Division, South African Medical Research Council, Cape Town, South Africa.

^7^Division of Chemical Pathology, Department of Pathology, Faculty of Medicine and Health Sciences, Stellenbosch University, Cape Town, South Africa.

^8^Division of Chemical Pathology, Department of Pathology National Health Laboratory Service, Tygerberg Hospital, Tygerberg, South Africa.

^9^South African National Department of Science and Innovation, South Africa.

^10^Department of Pathology, Division Medical Microbiology and Immunology, Faculty of Medicine and Health Sciences, Stellenbosch University, Tygerberg Hospital, Cape Town, South Africa.

^11^Centre for HIV and STIs, National Institute for Communicable Diseases, and Faculty of Health Sciences, University of the Witwatersrand, Johannesburg, South Africa.

^12^Wellcome Centre for Infectious Diseases Research in Africa, Institute of Infectious Disease and Molecular Medicine, University of Cape Town, Observatory 7925, South Africa.

^13^ Department of Infectious Diseases, Imperial College London, W12 0NN, United Kingdom

^14^The Francis Crick Institute, London, NW1 1AT, United Kingdom.

^15^Genomics Centre, South African Medical Research Council, Tygerberg, Cape Town, South Africa.

^16^Office of the President, South African Medical Research Council, Cape Town, South Africa

^17^Perinatal HIV Research Unit, Faculty of Clinical Medicine, University of the Witwatersrand, Chris Hani Baragwanath Academic Hospital, Johannesburg, South Africa.

^#^Authors contributed equally.

Corresponding author: Craig Kinnear, [gkin@sun.ac.za](mailto:gkin@sun.ac.za)

**Supplementary Figures.**

| **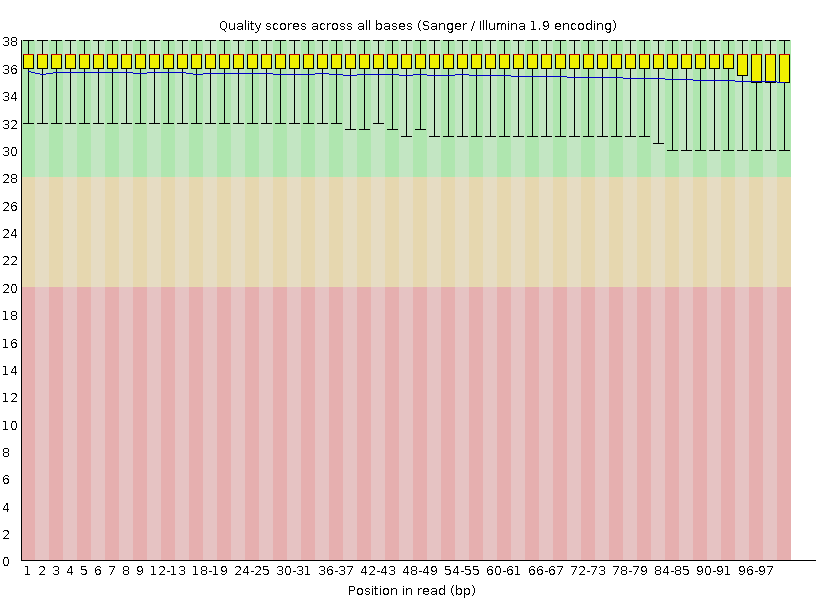**  **A** | 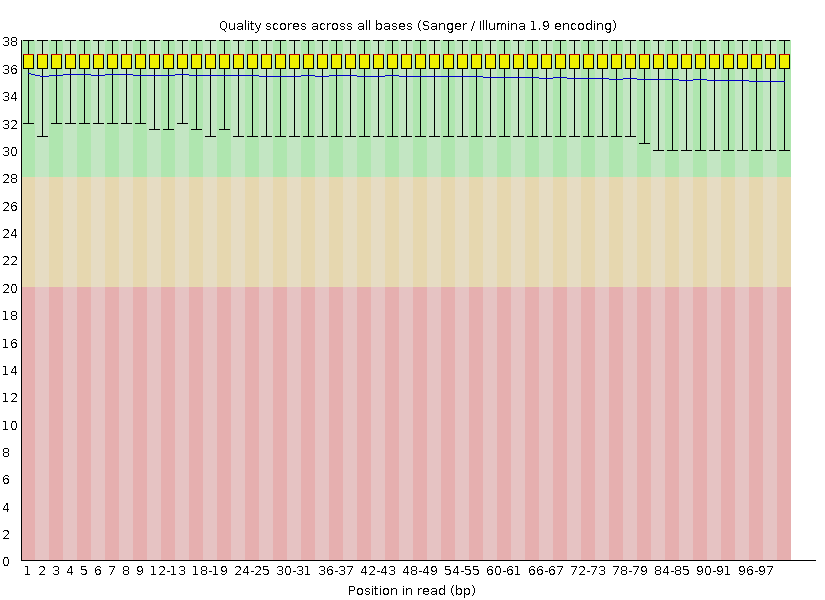  **B** |
| --- | --- |

**Supplementary Figure 1A and B.** Distribution of nucleotide quality parameters across forward (A) and reverse (B) reads for sample A sequenced at the Beijing Genomics Institute, China.

| **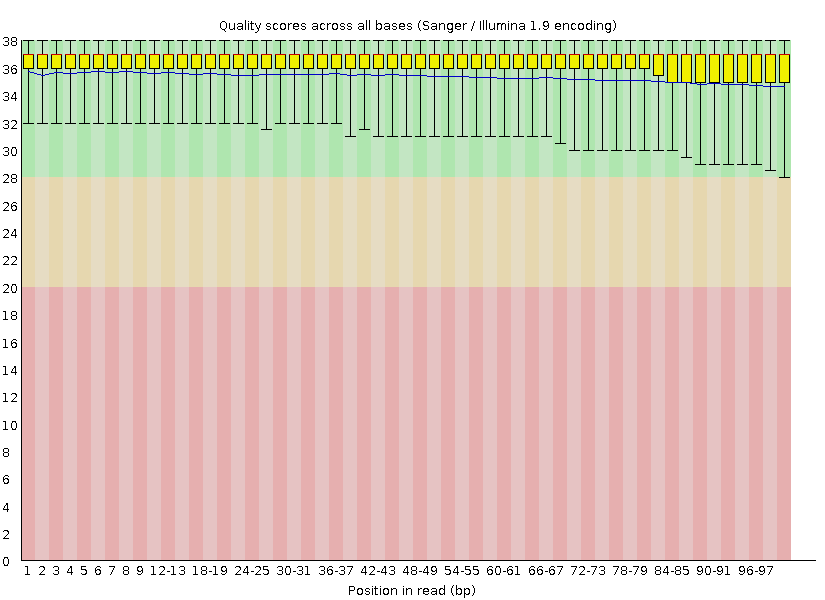**  **A** | 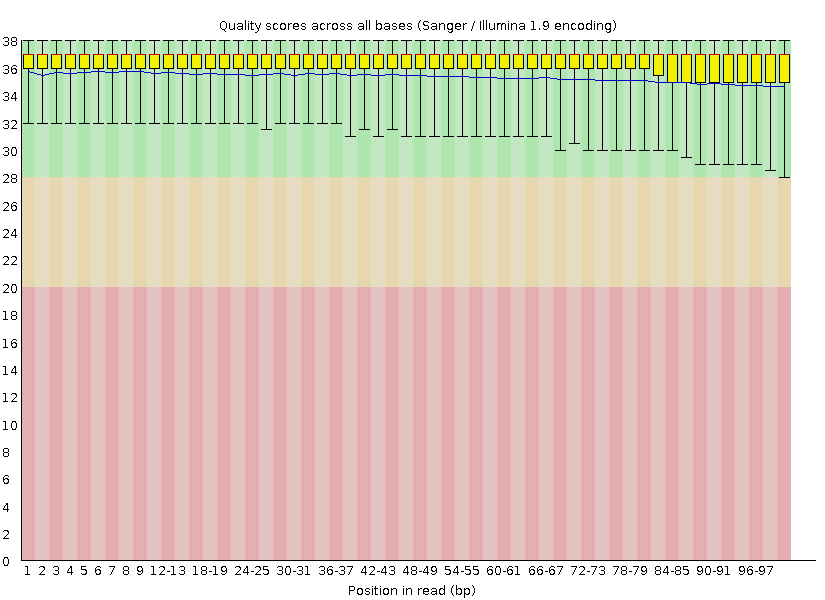  **B** |
| --- | --- |

**Supplementary Figure 2A and B.** Distribution of nucleotide quality parameters across forward (A) and reverse (B) reads for sample A sequenced at the Genomics Centre, South Africa.

| 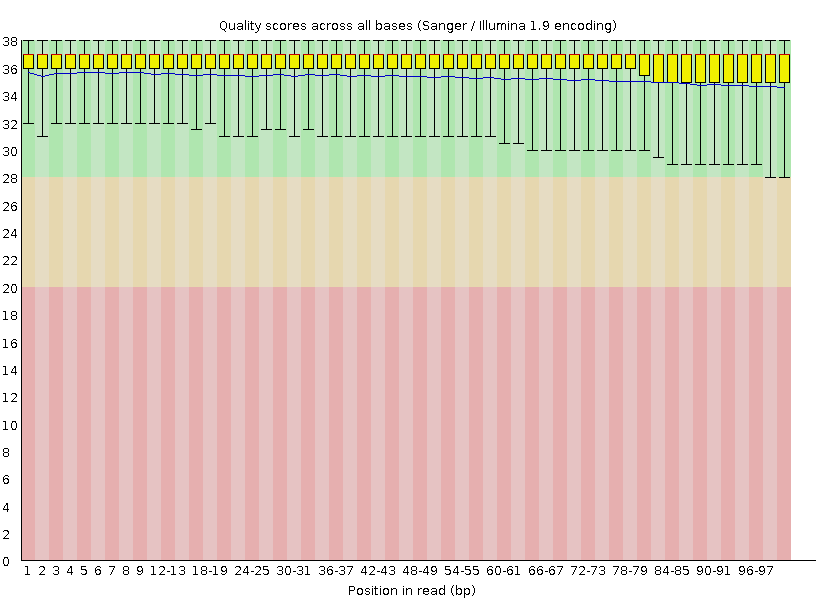  **A** | 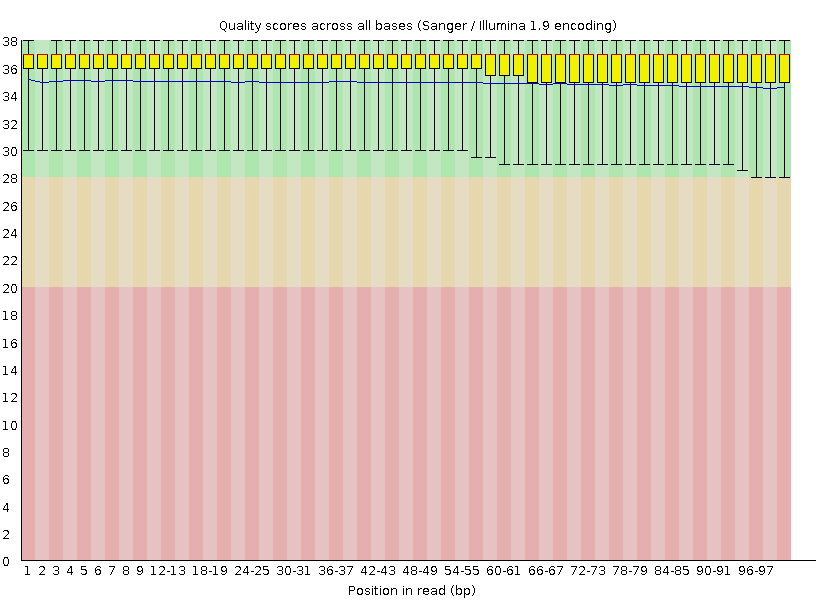  **B** |
| --- | --- |

**Supplementary Figure 3A and B.** Distribution of nucleotide quality parameters across forward (A) and reverse (B) reads for sample B sequenced at the Genomics Centre, South Africa.

| 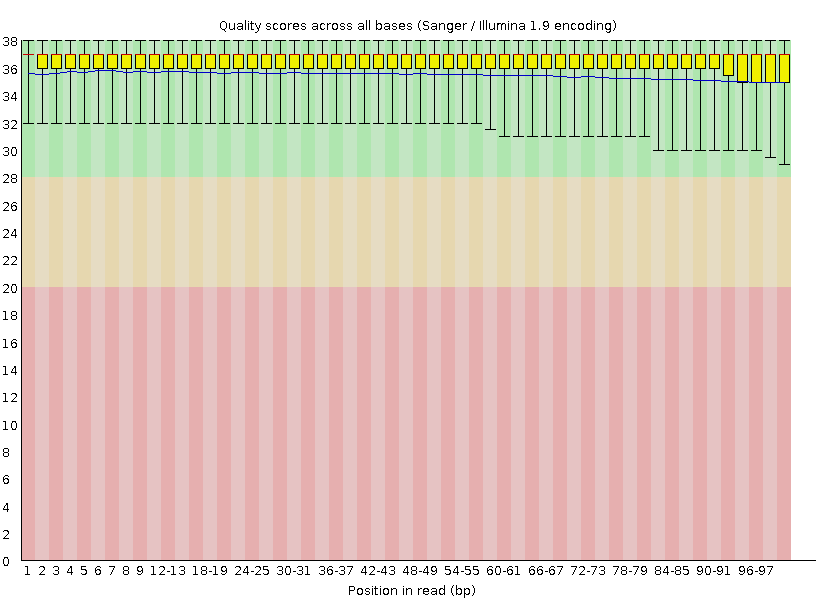  **A** | 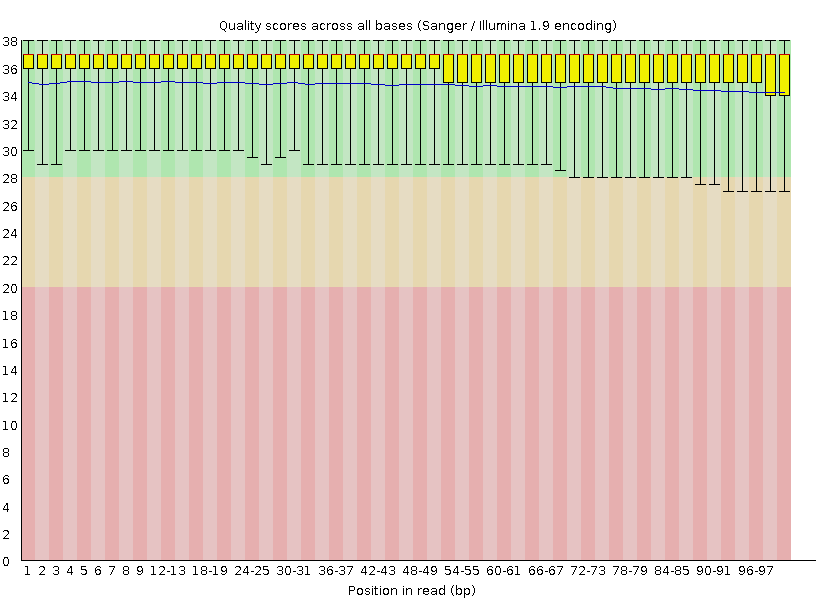  **B** |
| --- | --- |

**Supplementary Figure 4A and B.** Distribution of nucleotide quality parameters across forward (A) and reverse (B) reads for sample C sequenced at the Genomics Centre, South Africa.

| 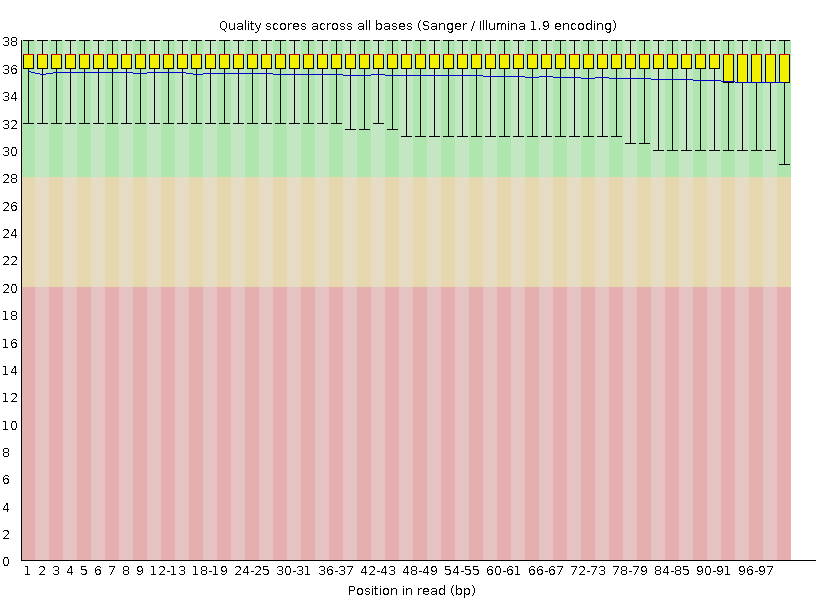  **A** | 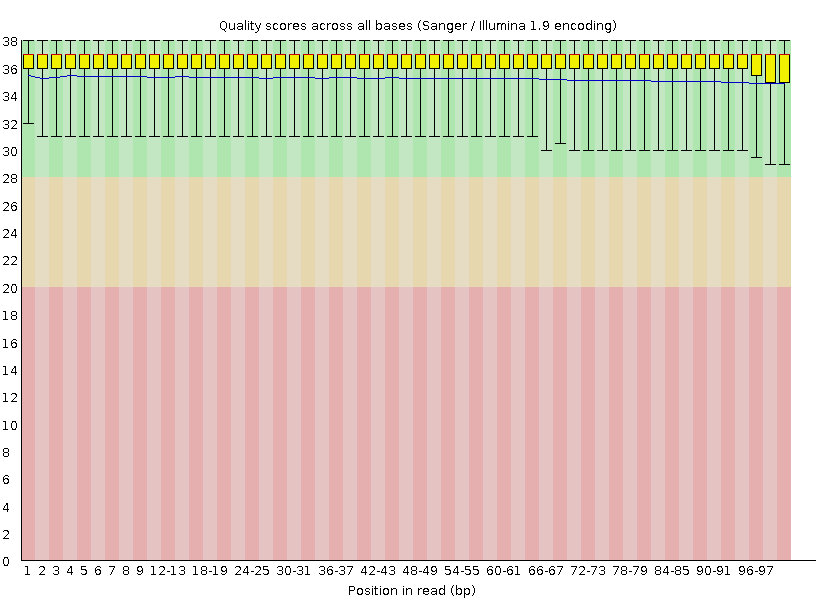  **B** |
| --- | --- |

**Supplementary Figure 5A and B.** Distribution of nucleotide quality parameters across forward (A) and reverse (B) reads for sample D sequenced at the Genomics Centre, South Africa.

| 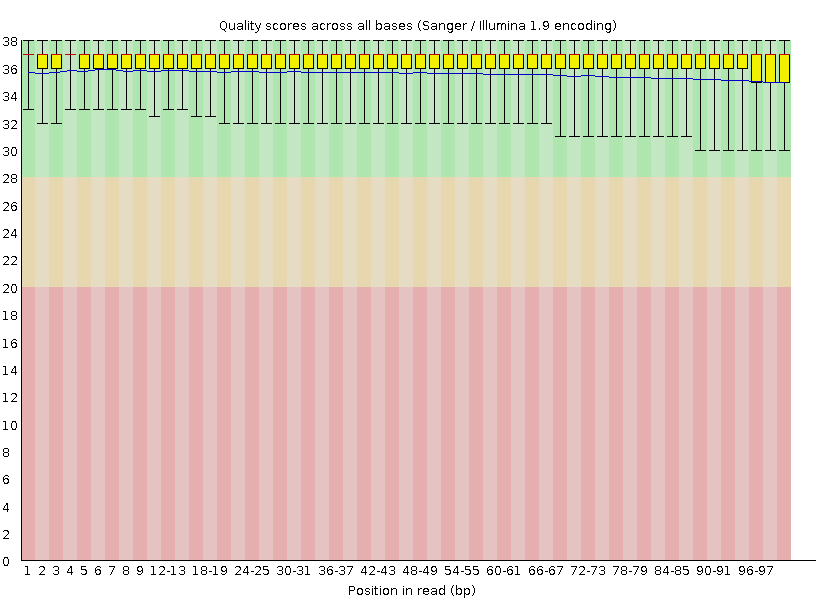  **A** | 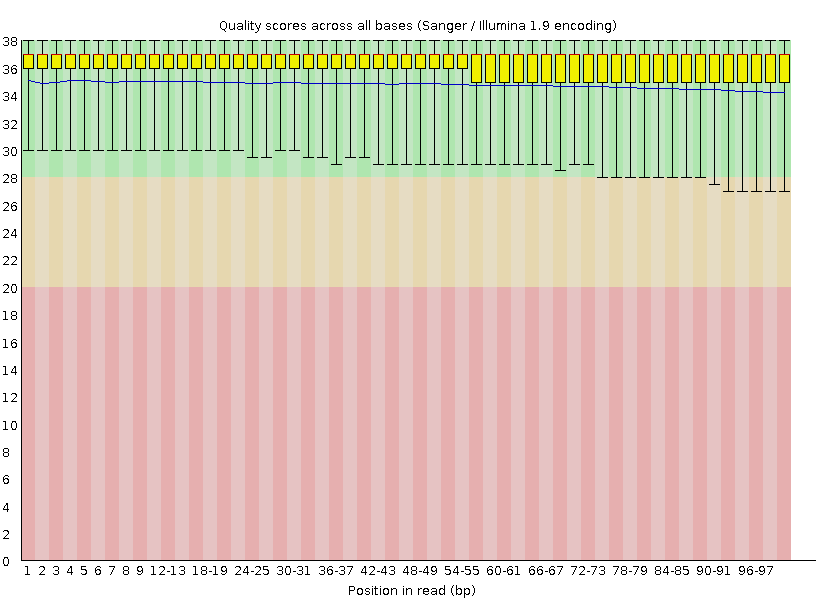  **B** |
| --- | --- |

**Supplementary Figure 6A and B.** Distribution of nucleotide quality parameters across forward (A) and reverse (B) reads for sample E sequenced at the Genomics Centre, South Africa.

| 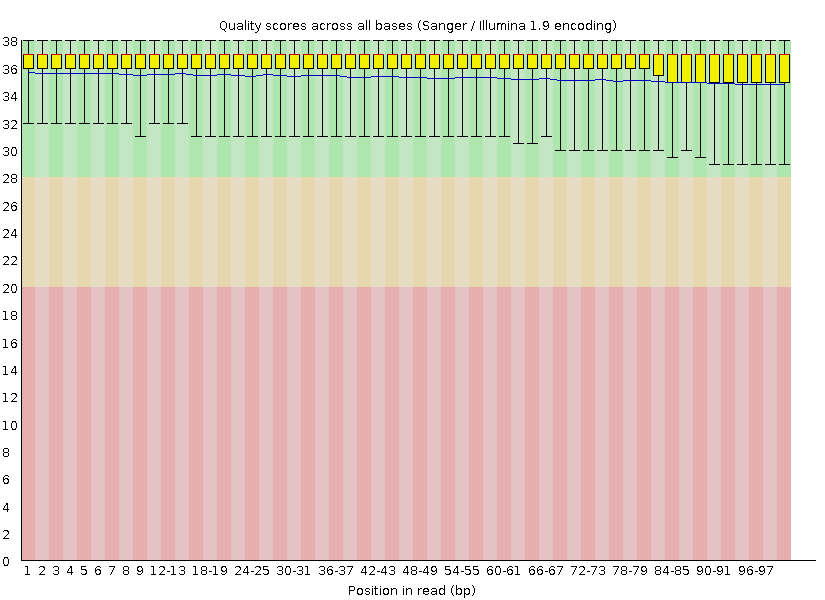  **A** | 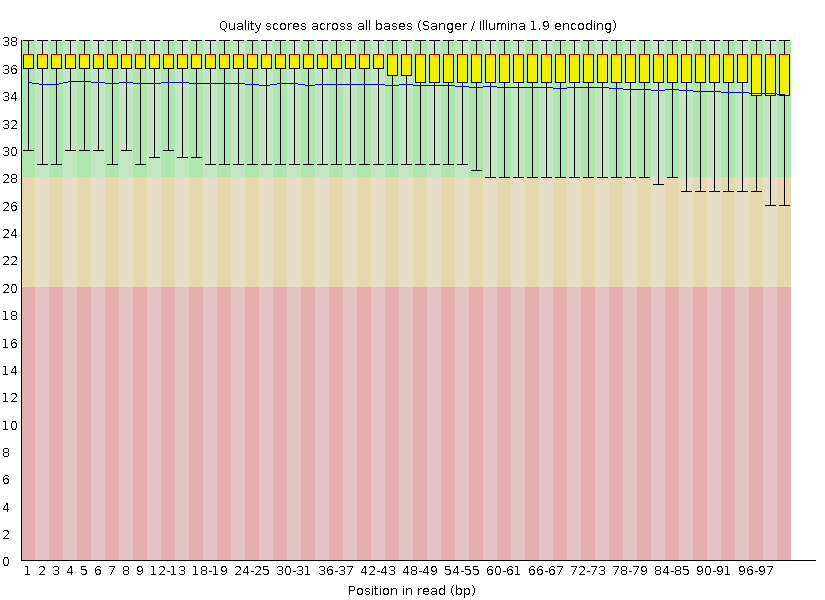  **B** |
| --- | --- |

**Supplementary Figure 7A and B.** Distribution of nucleotide quality parameters across forward (A) and reverse (B) reads for sample F sequenced at the Genomics Centre, South Africa.

.

**Supplementary Table 1: Summary of all variants identified in all 7 samples.**

| **Sample** | **A in China** | **A in South Africa** | **B** | **C** | **D** | **E** | **F** |
| --- | --- | --- | --- | --- | --- | --- | --- |
| **Instrument** | BGISEQ-500 | MGISEQ-2000 | MGISEQ-2000 | MGISEQ-2000 | MGISEQ-2000 | MGISEQ-2000 | MGISEQ-2000 |
| **Average coverage** | 36.32 | 36.41 | 36.79 | 36.91 | 36.32 | 36.41 | 36.23 |
| **Total number of variants** | **4,841,151** | **4,847,679** | **4,753,897** | **4,781,930** | **4,485,241** | **4,563,634** | **4,592,587** |
| **SNPs** | 3,907,017 | 3,913,468 | 3,815,771 | 3,842,348 | 3,531,385 | 3,623,269 | 3,636,764 |
| **Found in dbSNP** | 3,617,507 | 3,615,260 | 3,549,049 | 3,579,916 | 3,290,191 | 3,358,408 | 3,386,191 |
| **Novel** | 289,510 | 298,208 | 266,722 | 262,432 | 241,194 | 264,861 | 250,573 |
| **Homozygous** | 1,683,211 | 1,680,224 | 1,638,110 | 1,677,224 | 1,478,591 | 1,561,991 | 1,555,808 |
| **Heterozygous** | 2,223,806 | 2,233,244 | 2,177,661 | 2,165,124 | 2,052,794 | 2,061,278 | 2,080,956 |
| **Indels** | 934,134 | 928,211 | 938,126 | 939,582 | 953,856 | 940,365 | 958,823 |
| **Found in dbSNP** | 679,302 | 681,493 | 685,864 | 668,325 | 686,586 | 668,694 | 680,573 |
| **Novel** | 254,832 | 246,718 | 252,262 | 271,257 | 258,495 | 271,671 | 278,250 |
| **Homozygous** | 134,809 | 138,579 | 140,062 | 139,152 | 134,875 | 133,438 | 135,769 |
| **Heterozygous** | 799,342 | 789,632 | 798,064 | 800,430 | 818,981 | 806,927 | 823,054 |
